# Chemical Composition and Selective Bioactivities of *Piper platylobum* Sodiro Essential Oil

**DOI:** 10.3390/plants14213287

**Published:** 2025-10-27

**Authors:** Jairo Jaime-Carvajal, Nicole Pesántez, José Ballesteros, Vladimir Morocho, Omar Malagón

**Affiliations:** 1 Programa de Doctorado en Química, Universidad Técnica Particular de Loja, Calle Paris s/n y Praga, Loja 110107, Ecuador; 2 Grupo de Investigación en Aplicaciones Biotecnológicas, GIAB, Universidad Politécnica Salesiana, UPS, Carrera de Bioquímica y Farmacia, Campus María Auxiliadora, Kilómetro 19.5, Vía a La Costa, Guayaquil 090901, Ecuador; 3 Carrera de Bioquímica y Farmacia, Universidad Técnica Particular de Loja, Loja 110107, Ecuador; 4 Independent Researcher, Loja 110102, Ecuador; 5 Departamento de Química, Universidad Técnica Particular de Loja, Loja 110107, Ecuador

**Keywords:** dillapiole, acetylcholinesterase inhibition, antioxidant activity, antimicrobial activity

## Abstract

Essential oils from the genus *Piper* are recognized for their chemical diversity and biological potential, yet *Piper platylobum* has been scarcely investigated. This study aimed to characterize the chemical composition of the leaf essential oil of *P. platylobum* and evaluate its antimicrobial, antioxidant, and anticholinesterase activities. The oil was obtained by steam distillation and analyzed through gas chromatography–mass spectrometry (GC-MS) and gas chromatography equipped with a flame ionization detector (GC-FID), leading to the identification of 35 compounds that accounted for 91.11% of the volatile fraction. Dillapiole (42.0%) was the principal constituent, followed by α-(*E*)-bergamotene (5.69%), (*E*)-caryophyllene (5.01%), and (*E*)-isocroweacin (3.75%). Biological assays revealed selective antimicrobial activity, with inhibition observed only against *Enterococcus faecium* (MIC = 1000 µg/mL), while no effect was detected against other bacterial or fungal strains tested. Antioxidant evaluation showed moderate activity in the ABTS assay (SC_50_ = 335.71 ± 1.43 µg/mL; TEAC = 45.85 ± 1.68 µM Trolox/g EO), but no activity in the DPPH assay. The essential oil also displayed moderate inhibition of acetylcholinesterase (IC_50_ = 76.86 ± 1.00 µg/mL), suggesting a potential role in neuroprotective applications. This study constitutes the first report on the chemical composition and biological activities of *P. platylabum* essential oil, highlighting its potential as a novel source of bioactive compounds.

## 1. Introduction

Since ancient times, medicinal plants have played a fundamental role in human health, serving as the empirical basis for numerous traditional medical systems and as a primary source of pharmacologically active compounds [1,2]. It is estimated that over 50% of modern drugs are derived, directly or indirectly, from natural products, predominantly of plant origin [1,3]. This highlights the strategic importance of biodiversity as a reservoir of bioactive molecules with potential therapeutic and agroindustrial applications [4].

Among plant metabolites, essential oils (EOs) have attracted considerable attention in recent decades. These volatile and complex mixtures, primarily composed of terpenes and phenylpropanoids, are biosynthesized through specific pathways such as the mevalonate and methylerythritol phosphate pathways [5]. Beyond their structural diversity, EOs fulfill critical ecological functions, including defense against herbivores and pathogens, as well as the attraction of pollinators [6]. Their chemical composition varies widely according to genetic, environmental, and technological factors, which directly influence their bioactivity [7].

Numerous studies have demonstrated the pharmacological and agro-industrial potential of EOs, exhibiting antioxidant, antimicrobial, anti-inflammatory, insecticidal, and repellent activities [6,8,9]. These properties position EOs as promising natural candidates for the development of novel pharmaceuticals, biopesticides, and food preservatives, particularly in a global context where challenges such as antimicrobial resistance and pesticide contamination demand more sustainable solutions [10,11].

**Figure 1:**
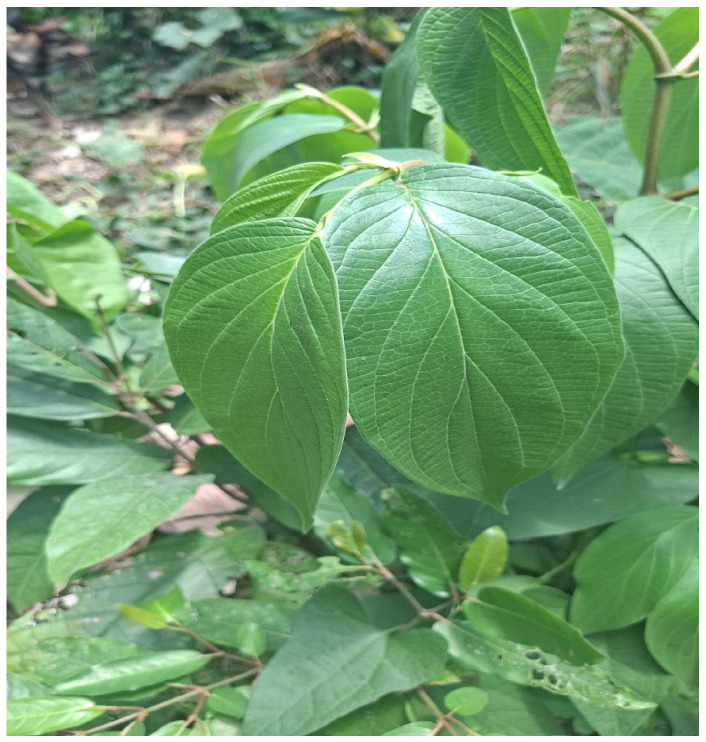
*P. platylobum.* collected in Ecuador. Photo provided by one of the authors (J.J.C).

Among botanical families of interest, Piperaceae—and specifically (Figure 1) the genus *Piper*—stands out due to its chemical richness and biological diversity. This genus comprises over 2000 species, primarily distributed in tropical regions, many of which have documented traditional uses for the treatment of respiratory, digestive, and inflammatory disorders [12,13]. Representative species such as *P. nigrum*, *P. betle*, and *P. longum* are well known for their ethnomedicinal value and bioactive compounds, including piperine, which exhibits antioxidant and anti-inflammatory properties [14,15]. In addition, various *Piper* species produce essential oils containing terpenes such as α-pinene, β-caryophyllene, and germacrene D, which have been associated with multiple biological activities [15]. Studies on species such as *P. aduncum* and *P. marginatum* have demonstrated their efficacy as antimicrobial, antioxidant, and natural pesticidal agents [16,17].

However, *P. platylobum* Sodiro. has been scarcely investigated, with an almost complete lack of published phytochemical and pharmacological data. Although several *Piper* species have been investigated, unexplored diversity still contrasts with the genus’s bioactive potential, reinforcing its relevance for scientific exploration and bioprospecting in tropical ecosystems [18]. In this context, the present study aims to characterize the essential oil extracted from the leaves of *P. platylobum* using gas chromatography–mass spectrometry (GC–MS) for qualitative analysis and gas chromatography with a flame ionization detector (GC–FID) for quantitative analysis, and to evaluate its biological potential in activities of relevance for pharmaceutical, food, and agricultural applications [19].

## 2. Results

### 2.1. Yield and Chemical Composition

**Figure 2:**
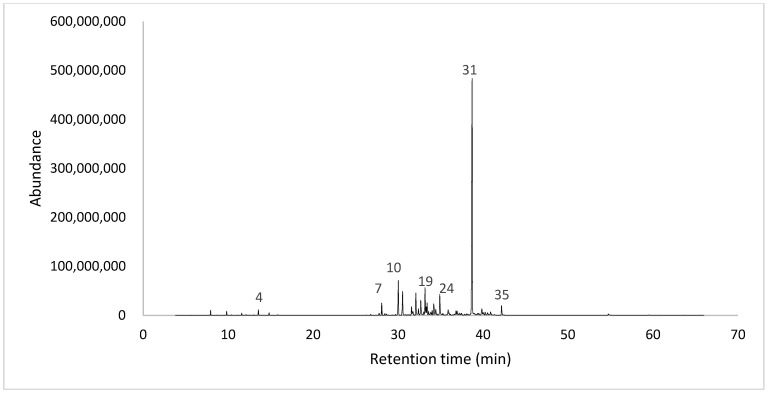
Total ion chromatogram of *P. platylobum* leaf essential oil.

The essential oil yield obtained from dried leaves of *P. platylobum* was 0.35% (*w*/*w*); the analysis by gas chromatography (GC) of the essential oil revealed a complex chemical profile, with the identification and quantification of thirty-five compounds (Table 1). Oxygenated phenylpropanoids accounted for 51.97% of the total, followed by hydrocarbon sesquiterpenes, which constituted 31.54%. Hydrocarbon monoterpenes and oxygenated sesquiterpenes were found in smaller concentrations, at 4.00% and 3.61%, respectively. Together, these results allowed the chemical identification of 91.11% of the total volatile compounds in the essential oil (Figure 2).

### 2.2. Antimicrobial Activity of Essential Oil

Based on Table 2, the essential oil extracted from the leaves of *P. platylobum* showed a targeted and specific antimicrobial activity against the panel of bacterial strains evaluated. The oil did not exhibit any activity against *Staphylococcus aureus* or *Pseudomonas aeruginosa*. However, a notable exception was recorded, a Minimum Inhibitory Concentration (MIC) of 1000 µg/mL against *Enterococcus faecium*, which suggests a moderate and specifically targeted inhibition of this strain.

### 2.3. Antioxidant Capacity

The DPPH assay was employed to assess the antioxidant capacity of *P. platylobum* essential oil. The results were compared with those obtained for the positive control Trolox (Table 3), which served as a standard reference due to its well-established antioxidant activity. The essential oil of *P. platylobum* exhibited moderate antioxidant activity, with values markedly lower than those of Trolox. This effect could not be attributed to its principal constituent, dillapiole, suggesting that the observed activity may result from synergistic interactions among different components or from the contribution of minor metabolites.

### 2.4. Anticholinesterase Activity

**Figure 3:**
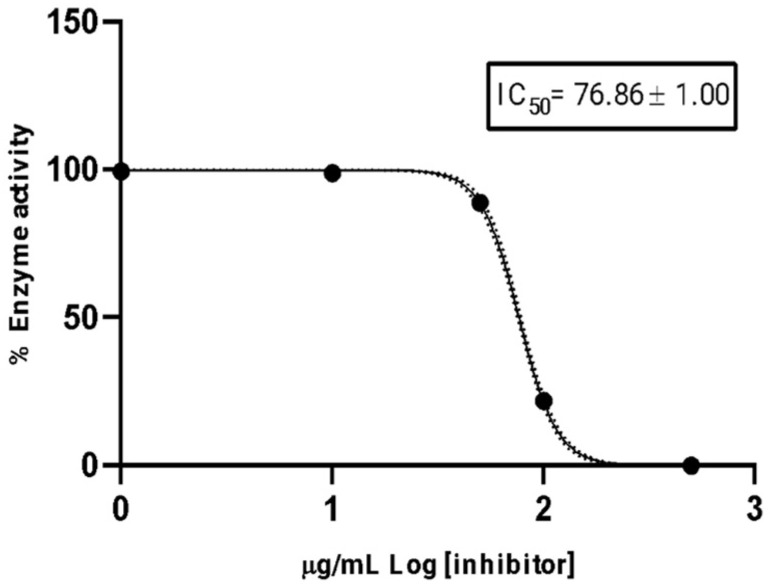
AChE inhibitory response from the *P. platylobum* essential oil expressed as IC_50_, calculated from non-linear regression curve data fitting analysis.

The essential oil of *P. platylobum* demonstrated a moderate inhibition of acetylcholinesterase, with an IC_50_ of 76.86 ± 1.00 µg/mL. In contrast, the reference inhibitor Donepezil exhibited a markedly higher potency, with an IC_50_ of 12.40 ± 1.35 µM (Figure 3).

## 3. Discussion

The essential oil yield obtained from the dried leaves of *P. platylabum* was relatively low (0.35% *w*/*w*). Previous studies have shown that some tropical *Piper* species can produce higher yields; for example, *P. xylosteoides* reached 1.8% (dry weight basis) [21]. In contrast, yields reported for other species are closer to those observed in the present work. Essential oils obtained from aerial parts of *P. capense*, *P. guineense* and *P. umbellatum* yielded 0.15%, 0.10%, and 0.13%, respectively, while *P. nigrum* fruits produced 0.26% [22]. This variation reflects the considerable intra- and interspecific diversity in essential oil production within the genus. Such differences are commonly attributed to geographical distribution, environmental conditions, and the occurrence of distinct chemotypes [23]. Consequently, the relatively low yield of *P. platylabum* is consistent with values reported for other *Piper* species and reinforces the importance of chemotypic and ecological factors when evaluating new taxa for essential oil bioprospecting.

The essential oil (EO) of *P. platylobum* contains more than 35 compounds, some of which are terpenes present at very low concentrations that together represent less than 40% of the essential oil content. The main component of *P. platylobum* EO is dillapiol (42.0%). Other vital elements of this essential oil include α-(*E*)-bergamotene (5.69%), (*E*)-caryophyllene (5.01%), and (*E*)-isocroweacin (3.75%). It also contains several sesquiterpenes, such as germacrene D, δ-amorphene, α-humulene, and bicyclogermacrene, all present at concentrations above 1%. Studies by Gomes da Camara et al. [24] and Pereira Filho et al. [25] reported dillapiole as the principal constituent of the essential oil from *P. aduncum* leaves, with relative abundances of 78.40% and 81.9%, respectively. However, contrasting results were reported in *P. corcovadense* by Fontoura et al. [26], who identified *trans*-sesquisabinene hydrate (24.91%), *trans*-caryophyllene (10.75%), β-pinene (5.61%), *trans*-β-farnesene (5.22%), 14-hydroxycaryophyllene (4.63%), limonene (3.76%), and *p*-cymene (3.62%) as the main constituents of the essential oil. Likewise, the essential oil of *P. rostratum* Roxb presented a different chemical profile, with γ-muurolene (14.1%), γ-cadinene (13.2%), allylpyrocatechol diacetate (11.5%), chavicol (8.2%), α-humulene (7.8%), and hydroxychavicol (6.9%) as the most abundant compounds [27]. In the case of *P. nigrum*, it was dominated by β-caryophyllene (18.64 ± 0.84%), limonene (14.95 ± 0.13%), sabinene (13.19 ± 0.17%), β-pinene (9.71 ± 0.12%), 3-carene (8.56 ± 0.11%), and α-pinene (7.96 ± 0.14%) [28]. These results confirm the remarkable chemical diversity within the genus *Piper*, where each species displays a unique volatile profile dominated by either sesquiterpenes or monoterpenes. In this context, dillapiole, a naturally occurring polyalkoxybenzene, has been consistently reported to exhibit antimicrobial activity [29]. Moreover, when present at high concentrations in the essential oils of other *Piper* species, dillapiole is considered one of the principal bioactive constituents contributing to their antimicrobial properties [30].

Evaluation of the antimicrobial activity of *P. platylobum* essential oil revealed selective efficacy, showing activity only against *Enterococcus faecium* with a MIC of 1000 µg/mL. The oil was not effective against other strains tested, including *Staphylococcus aureus*, *Staphylococcus epidermidis*, *Escherichia coli*, *Pseudomonas aeruginosa*, *Aspergillus niger*, and *Candida albicans*. Compared with related species, this activity appears relatively weak. For instance, *P. barbatum* essential oil demonstrated a broader antimicrobial spectrum, with strong inhibition (<500 µg/mL) against *S. aureus* (264 µg/mL), *Streptococcus mutans* (132 µg/mL), *Candida albicans* (132 µg/mL), and *Candida tropicalis* (264 µg/mL) [31]. Similarly, Houng et al. [32] reported that essential oils from leaves and stems of *Piper* species exhibited values of 16–64 µg/mL against Gram-positive bacteria (*S. aureus* and *B. cereus*), showing activities comparable or superior to streptomycin (128–256 µg/mL). These oils also inhibited *C. albicans* with 128 µg/mL, although they were less effective against Gram-negative bacteria such as *E. coli* and *P. aeruginosa*. Moreover, ethanolic extracts from *Piper* spp. have also shown remarkable antimicrobial potential; Alves et al. [33] reported MIC values <100 µg/mL against *Salmonella* spp. in maceration-derived extracts, while Soxhlet-derived samples exhibited broad inhibition against most microorganisms tested, except *P. aeruginosa*. Considering these findings, the antimicrobial performance of *P. platylobum* essential oil appears relatively modest compared with other *Piper* species. Such variability may be associated with differences in chemical composition, particularly the relative abundance of phenylpropanoids or oxygenated sesquiterpenes, which are often linked to higher antimicrobial potency [34]. Moreover, the biological activity of essential oils is not solely determined by the major component but often results from synergistic or antagonistic interactions among volatile constituents. In the case of *P. platylobum*, the predominance of dillapiole combined with relatively low levels of oxygenated sesquiterpenes or phenolic compounds—chemical groups often associated with higher antimicrobial potency—may account for the limited activity observed [35].

The AChE inhibitory activity of *P. platolobum* essential oil is reported for first time, showing a moderate effect with an IC_50_ value of 76.86 ± 1.00 µg/mL. Rezod et al. [36] found that the essential oil of *P. crassipes*, predominantly chavibetol (59.8%), exhibited moderate inhibitory effects on acetylcholinesterase (AChE) and butyrylcholinesterase (BChE), with IC_50_ values of 77.2 and 89.2 µg/mL, respectively. Salleh et al. [36] analyzed ten Malaysian *Piper* species, identifying *P. erecticaule* as the most potent, with 22.9% AChE inhibition. The essential oil of *P. platylobum* contains a variety of bioactive compounds that may contribute to acetylcholinesterase (AChE) inhibition. Among the monoterpenes, α-pinene has been reported to exhibit multiple biological activities, including AChE inhibition, antifungal activity, and natural insecticidal properties, and has been used for centuries in the production of flavors and fragrances [37]. Among them, β-caryophyllene is present at high levels and has been reported to possess potential AChE inhibitory activity. Similarly, sesquiterpenes and phenylpropanoids, including caryophyllene oxide, isospathulenol, (+)-spathulenol, β-bisabolene, and asaricin—which predominates in several *Piper* species—have demonstrated potent inhibition of AChE [38]. These findings support the notion that *Piper* essential oils, including that of *P. platylobum*, may serve as a valuable natural source of bioactive compounds with therapeutic potential for Alzheimer’s disease and other central nervous system disorders.

The essential oil of *P. platylabum* exhibited moderate radical scavenging capacity in chemical assays, with an SC_50_ value of 335.71 ± 1.43 µg/mL in the ABTS assay and a TEAC value of 45.85 ± 1.68 µM Trolox/g EO, while no activity was detected in the DPPH test. These results indicate a potential chemical antioxidant capacity, but they do not necessarily reflect biological antioxidant activity. Indeed, the DPPH and ABTS assays are based on single electron transfer or hydrogen atom transfer mechanisms that measure the ability of compounds to neutralize synthetic radicals in vitro, rather than in biological systems [39,40]. Similarly, Mata et al. explained that this trend may be due to the fact that the ABTS method is particularly suitable for evaluating lipophilic antioxidants and is therefore more appropriate for assessing the antioxidant potential of essential oils. In contrast, the lack of activity in the DPPH assay may be related to the limited hydrogen-donating capacity of terpenes and their low solubility in methanol, the solvent used in this test [41]. These results are consistent with previous findings for *P. ecuadorense*, whose essential oils, mainly composed of sesquiterpenes and monoterpenes, also displayed higher activity in the ABTS assay compared to DPPH [42]. Other studies support this idea: *P. cubeba* oil, for example, was shown to be superior to *P. nigrum* oil in its antioxidant [43]. Similarly, the essential oil of *Piper betle* exhibits strong antioxidant activity in the DPPH assay, with radical scavenging values ranging from 72% to 89%, depending on the drying method used [44]. When comparing these findings with other members of the genus, clear differences emerge. P. acutifolium displayed stronger radical-scavenging activity, with IC_50_ values of 160.12 ± 0.30 µg/mL in the DPPH assay, 138.10 ± 0.06 µg/mL in ABTS, and 450.10 ± 0.05 µg/mL in FRAP [45], suggesting a broader antioxidant potential than that observed for *P. platylabum.* Moreover, Rodríguez et al. [46] reported even more pronounced antioxidant capacity in the DPPH assay for *P. aduncum* (30.1 ± 1.8 µg/mL), *P. auritum* (14.8 ± 0.5 µg/mL), and *P. umbellatum* (32.3 ± 1.3 µg/mL), values that are substantially lower than those obtained for *P. platylabum* in the ABTS test. These findings in other *Piper* species reinforce the notion that environmental and developmental factors can influence the chemical composition and thus bioactivity of *P. platylobum.* Given that there are no studies addressing how ecological conditions affect *P. platylobum* essential oil, the results presented in the paper are a unique snapshot of its potential. This highlights the need for further research to explore how geographical location, climate, and plant maturity could alter its chemical profile and, consequently, its therapeutic and ecological properties [44]. Such studies would provide a more complete picture of the species’ potential as a source of bioactive compounds.

## 4. Materials and Methods

### 4.1. Plant Material

**Figure 4:**
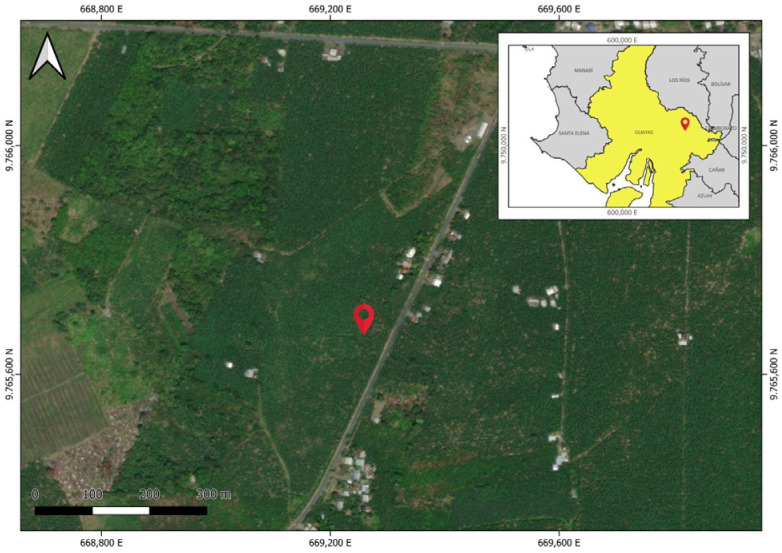
Geographic map showing the sampling site for the collection of *P. platylobum* in the tropical dry forest.

The leaves were collected at coordinates 2°07′09.5″S and 79°28′41.0″W (Figure 4), approximately 15 km southwest of the urban center of Milagro city, in Guayas Province. The site corresponds to a disturbed area of seasonal lowland evergreen forest located along the coastal region. This area is part of the Tumbes region of endemism, known for its high biodiversity and unique flora.

### 4.2. Essential Oil Isolation

The essential oil was extracted from 87.20 g of dried leaves which were dried in an electric dryer (model DY-330H, Lassele, Ansan City, Gyeonggi-do, Republic of Korea) at 35 °C for 48 h; the extraction process was carried out by steam distillation for 150 min. Once obtained, the essential oil was stored in a refrigerator at 4 °C until subsequent chemical and biological analysis, the plant material from was distilled in three repetitions.

### 4.3. Identification and Quantification of Essential Oil

#### 4.3.1. Qualitative Analysis

For qualitative analysis, a Thermo Fisher Scientific (Waltham, MA, USA) gas chromatograph (Trace 1310 series model) coupled to a mass spectrometry detector (ISQ 7000 brand) was used. An apolar TR-5MS capillary column with a 5% phenyl (equiv) polysilphenylene-siloxane stationary phase (30 m length × 0.25 mm internal diameter, and a film thickness of 0.25 μm) was used. The injection conditions were a temperature of 230 °C, with a split radius ratio of 1:80. Helium was used as a carrier gas, with a constant flow of 1 mL/min. The initial operating conditions of the oven were 50 °C for 3 min, followed by a ramp of 3 °C/min until reaching 230 °C, maintaining this final temperature for 3 min. The detector used was a single quadrupole mass spectrometer, operating in 0.2 scan mode with a mass range of 40–400 *m*/*z*, and temperatures of the transfer line of 250 °C and 230 °C for the ion source.

The components of the essential oil of *P. platylobum* were identified by comparing their mass spectra with those of reference compounds showing similar Linear Retention Indices (LRIs) reported in the literature [20]. LRIs were calculated according to the method of Van Den Dool and Kratz [41], based on the retention times of a homologous series of n-alkanes (C9–C22, TPH-6RPM, Chem Service) analyzed under the same GC conditions, to increase the reliability of the identification. Data acquisition and processing were managed via the Chromeleon XPS software, version 7.2.10 (Waltham, MA, USA), and mass spectral matching was performed using the NIST 17 MS from the internal chromatogram database. The compound was considered identified if the calculated retention index did not differ by ±25 from the reference values [47].

#### 4.3.2. Quantitative Analysis

Quantitative analysis was performed using gas chromatography coupled with a flame ionization detector (GC-FID). The same column and injection conditions were used as those used for gas chromatography–mass spectrometry (GC-MS). The relative amounts of the compounds were determined by GC-FID through peak area integration, without applying any correction factors.

### 4.4. Antimicrobial Activity

Antimicrobial activity was assessed using the antimicrobial potential and the protocol described by Cartuche et al. [48]. The antimicrobial potential and minimum inhibitory concentration (MIC) were determined using the broth microdilution technique and the double serial dilution system to obtain concentrations of 4000 to 31.25 µg/mL for each of the diluted wells. Gram-positive bacteria *(Enterococcus faecium* ATCC^®^ 27270, *Staphylococcus aureus* ATCC^®^ 25923, *Staphylococcus epidermidis* ATCC^®^ 12228) and two Gram-negative bacteria *(Escherichia coli* (O157:H7) ATCC^®^ 43888, *Pseudomonas aeruginosa* ATCC^®^ 10145) were selected. Antifungal activity was also evaluated *(Aspergillus niger* ATCC^®^ 6275, *Candida albicans* ATCC^®^ 10231). All these microorganisms were reactivated at a standard McFarland scale of 0.5 with an approximate of 1.5 × 10^8^ colony-forming units per milliliter (CFU/mL). Cation-adjusted Mueller–Hinton II (MH II) assay media were used for bacteria, and Sabouraud broth for fungi. Commercial antimicrobial products were used as positive controls: for Gram-positive bacteria, *E. faecium*, *S. aureus*, and *S. epidermidis*, ampicillin solution (1 mg/mL) was used; for Gram-negative bacteria such as *E. coli* and *P. aeruginosa*, ciprofloxacin (1 mg/mL) was used; finally, amphotericin B (250 µg/mL) was used for the two fungi *A. niger* and *C. albicans.*

### 4.5. Antioxidant Capacity

Antioxidant activity was assessed using the ABTS and DPPH radical scavenging assay, employing the methodology described by Cartuche et al. [48]. The ABTS assay was carried out by mixing ABTS (455 µM) with potassium persulfate (2596 µM) dissolved in 100 mL of ultrapure water, followed by continuous stirring for 12 h. The standard solution was adjusted in methanol to achieve an absorbance of 1.1 ± 0.02, measured at 734 nm using an EPOCH 2 microplate reader (BIOTEK, Winooski, VT, USA). For the DPPH assay, a working solution was prepared using the free radical 2,2-diphenyl-1-picrylhydrazyl (DPPH), 4.6 mg of DPPH dissolved in 100 mL of methanol. Subsequently, 270 µL of the working solution and 30 µL of the oil sample were placed in the 96-well plate and monitored at 515 nm for 60 min. Finally, Trolox reagent was used as a positive control and methanol as a blank control. These assays were expressed as SC 50 (radical scavenging concentration at 50%); both assays were performed in triplicate to ensure experimental accuracy.

### 4.6. Cholinesterase Assay

The inhibitory activity was carried out by the enzyme acetylcholinesterase (AChE) and was evaluated with a sample of essential oil of *P. platylobum*. In vitro evaluation was performed according to the method described by Ellman et al. [49], where the AChE enzyme from Electrophorus electricus (Sigma Aldrich, St. Louis, MO, USA) was used; briefly, 20 μL of acetylcholine (ATCh) was placed together with 60 μL of PBS buffer pH 7.4, 100 μL of Ellman’s reagent solution in Tris buffer (DNTB) and 20 ul of the analyzed sample, after which it was preincubated for 3 min at 25 °C and then 20 ul of enzyme solution of 0.5 U/mL was added to start the reaction. The reaction was placed in an EPOCH 2 microplate reader, and the released reaction product was monitored at 405 nm for 60 min.

The essential oil was prepared by dissolving 10 μL of oil in 990 μL of MeOH, and from this, eight 2-fold dilutions were made to obtain final concentrations of 8000, 4000, 2000, 1000, 500, 250, 125, and 62.5 μg/mL. Finally, the IC_50_ value was calculated from the progression curves using GraphPad Prism software (nonlinear regression analysis, PRISM 8.0.1, GraphPad, San Diego, CA, USA), and Donepezil hydrochloride was used as a positive control, with an IC_50_ value of 12.40 ± 1.35 nM, while MeOH served as a negative control.

## 5. Conclusions

The essential oil of *Piper platylobum* was investigated, a species that has received little scientific attention to date. The chemical analysis identified Dillapiole as the main constituent, and the biological assays showed that the oil has a weak but notable antimicrobial effect on *Enterococcus faecium* and a moderate anticholinesterase activity. The lack of activity in other assays suggests a specific biological profile for this species. This research contributes to the bioprospecting of tropical ecosystems and provides a foundation for future studies to isolate and characterize the bioactive compounds responsible for the observed activities. Further research should focus on fractionating the essential oil to pinpoint the specific molecules responsible for its anticholinesterase and antimicrobial effects, which could lead to the development of new pharmaceuticals or biopesticides.

## Figures and Tables

**Table 1:** Chemical composition of *P. platylobum* essential oil from Ecuador.

No.	RT	Compound	LRI Calculate	LRI Literature	Media ± SD	MF
1	7.94	α-Pinene	932	932	0.95 ± 0.05	C_10_H_16_
2	9.83	β-Pinene	979	974	0.93 ± 0.05	C_10_H_16_
3	11.60	α-Terpinene	1019	1014	0.41 ± 0.02	C_10_H_16_
4	13.55	γ-Terpinene	1062	1054	1.14 ± 0.07	C_10_H_16_
5	14.82	Terpinolene	1089	1086	0.57 ± 0.04	C_10_H_16_
6	27.77	α-Ylangene	1370	1374	0.45 ± 0.00	C_15_H_24_
7	28.08	α-Copaene	1377	1374	2.33 ± 0.09	C_15_H_24_
8	28.43	β-Bourbonene	1385	1387	0.36 ± 0.09	C_15_H_24_
9	30.02	(*E*)-Caryophyllene	1422	1417	5.01 ± 3.86	C_15_H_24_
10	30.53	α-(*E*)-Bergamotene	1434	1432	5.69 ± 1.18	C_15_H_24_
11	31.59	α-Humulene	1460	1452	1.52 ± 1.22	C_15_H_24_
12	31.75	allo-Aromadendrene	1464	1458	1.09 ± 0.91	C_15_H_24_
13	32.09	Croweacin	1472	1457	3.01 ± 1.92	C_11_H_12_O_3_
14	32.40	γ-Muurolene	1479	1478	1.97 ± 1.51	C_15_H_24_
15	32.67	Germacrene D	1486	1480	2.50 ± 1.15	C_15_H_24_
16	33.02	α-Amorphene	1494	1483	2.66 ± 0.86	C_15_H_24_
17	33.17	n-Pentadecane	1498	1500		C_15_H_32_
18	33.29	Bicyclogermacrene	1501	1500	0.93 ± 0.10	C_15_H_24_
19	33.41	α-Muurolene	1504	1500	3.74 ± 0.10	C_15_H_24_
20	33.59	(*E*,*E*)-α-Farnesene	1508	1505	0.19 ± 0.13	C_15_H_24_
21	33.85	Asaricin	1515	1495	0.41 ± 0.18	C_11_H_12_O_3_
22	34.01	γ-Cadinene	1519	1513	0.71 ± 0.10	C_15_H_24_
23	34.19	δ-Amorphene	1524	1511	1.41 ± 0.12	C_15_H_24_
24	34.90	(*E*)-Isocroweacin	1541	1553	3.75 ± 0.09	C_11_H_12_O_3_
25	35.27	α-Calacorene	1551	1544	0.34 ± 0.05	C_15_H_20_
26	35.89	Elemicin	1566	1555	0.72 ± 0.02	C1_2_H_16_O_3_
27	36.01	(*E*)-Nerolidol	1569	1561	0.57 ± 0.02	C_15_H_26_O
28	36.82	Spathulenol	1590	1577	0.77 ± 0.10	C_15_H_24_O
29	36.95	Caryophyllene oxide	1593	1582	1.30 ± 0.23	C_15_H_24_O
30	37.47	Guaiol	1607	1600	0.41 ± 0.07	C_15_H_26_O
31	38.71	Dillapiole	1640	1620	42.00 ± 1.53	C_12_H_14_O_4_
32	39.86	α-Cadinol	1670	1652	0.55 ± 0.35	C_15_H_26_O
33	40.25	Elemol acetate	1681	1680	0.47 ± 0.11	C_17_H_28_O_2_
34	40.53	Cadalene	1688	1675	0.64 ± 0.29	C_15_H_18_
35	40.89	(*E*)-Asarone	1698	1675	1.62 ± 0.10	C_12_H_16_O_3_
		Hydrocarbon monoterpenes (HM)			4.00	
		Hydrocarbon sesquiterpenes (HS)			31.54	
		Oxygenated sesquiterpenes (OS)			3.61	
		Other compounds			51.97	
		Total identified			91.11	

LRI_cal_: Calculated linear retention index; LRI_lit_: Linear retention index from Adams [20]; SD: mean standard deviation over three determinations; MF: molecular formula.

**Table 2:** Antibacterial and antifungal capacity of *P. platylobum* essential oil.

Microorganisms	*P. platylobum* essential Oil †	Antimicrobial Agent (Positive Control) †
**Cocci Bacteria**		**Ampicillin (1 mg/mL)**
*Enterococcus faecium* ATCC^®^ 27270	1000	<0.3906
*Staphylococcus aureus* ATCC^®^ 25923	Non active	<0.3906
*Staphylococcus epidermidis* ATCC^®^ 12228	Non active	<0.3906
**Rod-shaped Bacteria**		**Ciprofloxacin (1 mg/mL)**
*Escherichia coli* (O157:H7) ATCC^®^ 43888	Non active	1.5625
*Pseudomonas aeruginosa* ATCC^®^ 10145	Non active	<0.3906
**Yeasts and sporulated fungi**		**Amphotericin B (250 µg/mL)**
*Aspergillus niger* ATCC^®^ 6275	Non active	<0.098
*Candida albicans* ATTC^®^ 10231	Non active	<0.098

† MIC values are given as µg/mL.

**Table 3:** Half scavenging capacity (SC_50_) of *P. platylobum* essential oil.

Essential oil	ABTS	DPPH	TEAC
	**SC_50_ ± SD** (µg/mL—µM *)		**µM Trolox/g EO**
*P. platylobum*	335.71 ± 1.43	-	45.85 ± 1.68
Trolox *	29.09 ± 1.05	35.54 ± 1.04	-

* Trolox was used as a positive reference, and its values are given in µM. (-) Not active at the highest dose tested (8000 µg/mL).

## Data Availability

All data presented in this study are available in this article.
